# Supplementary material for: Splice-Junction-Based Mapping of Alternative Isoforms in the Human Proteome
Source: Cell Rep. Author manuscript; Available in PMC 2020 Jan 15. (PMC6961840; doi:10.1016/j.celrep.2019.11.026)
Supplement: 2 [file NIHMS1546469-supplement-2.zip › DF1/PXD010154/Heart/Heart_31_MYBPC3_AITQLLCETEGR.pdf]

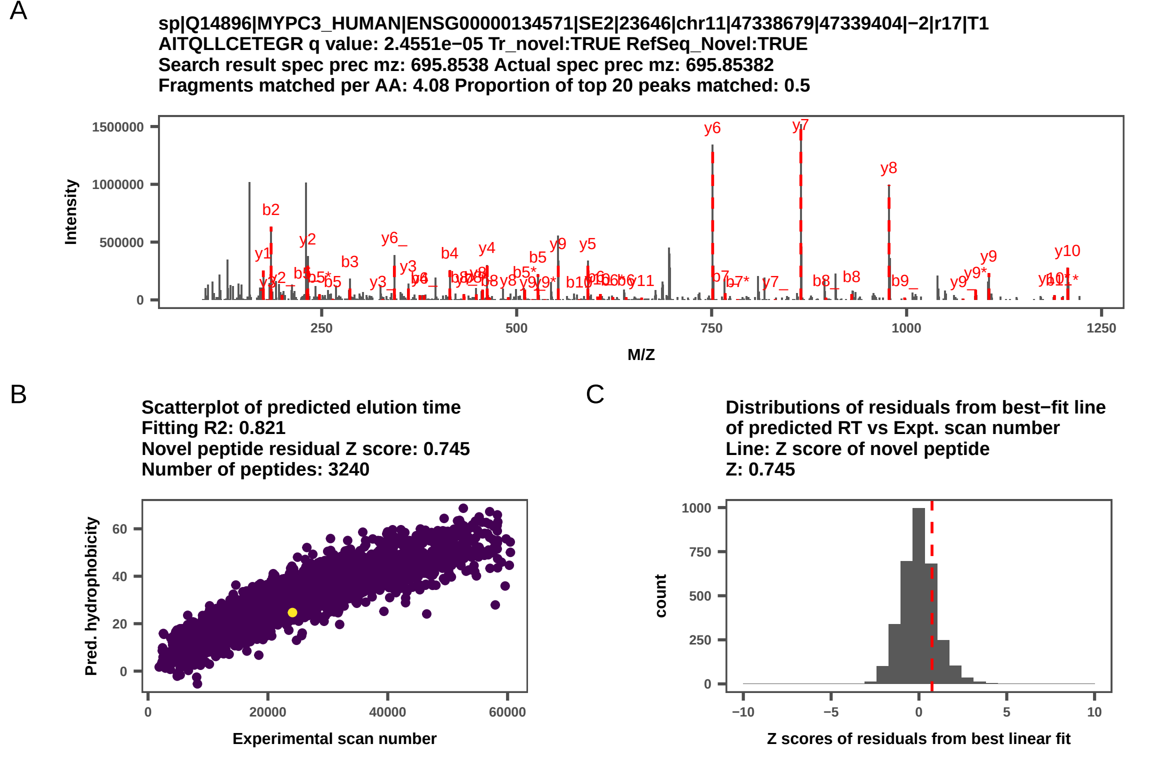
A
sp|Q14896|MYPC3_HUMAN|ENSG00000134571|SE2|23646|chr11|47338679|47339404|−2|r17|T1
AITQLLCETEGR q value: 2.4551e−05 Tr_novel:TRUE RefSeq_Novel:TRUE
Search result spec prec mz: 695.8538 Actual spec prec mz: 695.85382
Fragments matched per AA: 4.08 Proportion of top 20 peaks matched: 0.5
Intensity
1500000
1000000
500000
0
M/Z
250
500
750
1000
1250
y6
y7
y8
b2
y1
y2
b3
y6_
b4
y4
y3
b5
y9
y5
y9
y10
B
Scatterplot of predicted elution time
Fitting R2: 0.821
Novel peptide residual Z score: 0.745
Number of peptides: 3240
Pred. hydrophobicity
60
40
20
0
Experimental scan number
0
20000
40000
60000
C
Distributions of residuals from best−fit line
of predicted RT vs Expt. scan number
Line: Z score of novel peptide
Z: 0.745
count
1000
750
500
250
0
Z scores of residuals from best linear fit
−10
−5
0
5
10
